# 

**Published:** 1887-06-04

**Authors:** 


### The Hospital.

**An Institution, Family, and Parochial Journal of Hospitals, Asylums, and all Agencies for the Care of the Sick, Criticism and News.**

SATURDAY, JUNE 4TH, 1887.

### THE CHILDREN'S CORNER.

**Third Quarterly Prize Competition.**
Began 2nd Apr., 1887 ; ends 25th June, 1887.

**Puzzles—Tenth Week.**

No. 39. GEOGRAPHICAL CHARADE. My first is half of danger, my second and third together imply an indefinite number, and my whole is one of the principal countries in Europe.

No. 40. ARITHMETICAL PUZZLE. Divide thirteen into two equal numbers without a fraction.

* * * *
* * * * No. 41. SQUARE WORD. 1. A flower. 2. A king
* * * * of Mercia. 3. Acid. 4. Part of the head.
* * * *

No. 42. VEGETABLE ANAGRAMS. 1. Soon in. 2. So to a pet.

**Letters—Tenth Week.**

The Letter-subject for next week will be "Birds' Eggs."

**Results of Eighth Week—Puzzles.**

No. 31. CONUNDRUM. Her mitts = hermits.
No. 32. ENIGMA. Faith, Hope, and Charity.
No. 33. BIRDS' NAMES ENIGMATICALLY EXPRESSED. Swift, Kingfisher, Sparrow, Albatross.
No. 34. DOUBLE MESOSTICH.

E
E X e
E X C E L
D E e
L

*All right*—A. C. K., Agatha, Alsie, Bohemian Girl, Dot, R. Epton, Gom, Joe, A. G. S. McCulloch, Mikado, Nordisa, Scrooge, Skye; *Three right*—Tim, Violin, Umslopogaas, The Eda; *Two right*—Ellie, Peeler; *One right*—Etta.

**Letters.**

"Tell me," once said a philosopher, "whether a man loves plants, whether he takes a pleasure in their culture and in his garden, and I will tell you the manner of man he is" Those who love flowers and delight to take part in their cultivation, are, we may depend upon it, those who love to make their homes happy homes, and those around them participators in the pleasures they enjoy. Skye writes me the following sensible letter:

DEAR MR. EDITOR,—I think no home is complete without a garden. Everyone seems so pleased to see the seeds coming up, and then the lovely flowers after a while. I think working men show great taste in gardening. The Scotch are very good gardeners. I think Drummond Castle gardens, in Perthshire, are the finest I ever saw. They were laid out in the Italian style, with a great many splendid statues, fountains, and grottoes. I do not think a person *could* be wicked who spent his leisure time in gardening: and then it has the honour of being the first trade. I like old-fashioned gardens best, with plenty of roses and wallflowers, and with paths that you are not afraid to walk on for fear you might disarrange the gravel, like some gardens I know, where nothing takes *root*, but everything is put *in* when it is just beginning to bloom, and pulled out again whenever its gets withered, to make room for a fresh supply. I should not like such a garden.—Yours faithfully, SKYE, aged 13.
4, Milton Terrace, New Road, Chatham.

Violin writes me:—

DEAR MR. EDITOR,—I am going to tell you about my garden. We have a very big garden, and each of us has got a part of it. In the middle of mine I have a red rose-tree: it is bordered with poppies. I like gardening very much: we have a lot of seeds coming up. I am going to try to make a rockery at the back. I am gathering stones for it.
Yours faithfully, VIOLIN, aged 11.
Cavendish House, Colwyn Bay,

Etta writes a nice letter about her grandmamma's garden. Mikado and Bohemian Girl lament a lovely garden they once had, before they became the denizens of a big town. Ellie tells me she is training some plants for a prize competition. Peeler seems to be an ardent admirer of the floral beauties of nature; in a most interesting letter he tells me how he should like to see all the little pale-faced children of London transported to the green fields, just now in all their primal loveliness. The Eda writes me an excellent letter about a garden they visited two summers ago. I am very sorry to hear that Ada is unwell.

One mark each:—Bohemian Girl, Ellie, Mikado, Peeler, Skye, The Eda, Etta, Violin.

**Notices to Correspondents.**

MASTER A. DALE.—Thank you very much for your letter.

---

Answers, having the actual name and address (to which a pseudonym may be added for publication), must have "The Children's" Coupon attached (see advertisement pages), and be addressed to the "PUZZLE EDITOR," THE HOSPITAL, Norfolk House, Norfolk Street, Strand, W.C., not later than Thursday next.

### AMUSEMENTS WITH PROFIT.

### PRIZE COMPETITIONS.

**Quarterly Prize Competition.**
Began 23rd April; ends 16th July.

**No. 13. Double Acrostic.**

1. A linen vestment.
2. A province of British India.
3. A river of British Guiana.
4. A county of Scotland.
5. A celebrated mathematician and natural philosopher.
6. A colourless liquid.
7. One of the Society Islands.
8. A city in Germany celebrated for its University.

The primals and finals give the names of two celebrated doctors.

**No. 14. Heroism.**

Give a definition of "Heroism" in prose or verse; answers not to exceed one hundred words.

**Answers.**

No. 9. DOUBLE ACROSTIC.

| | | |
|---|---|---|
| B | rie | F |
| E | n | O |
| L | ende | R |
| F | ee | S |
| A | delph | I |
| S | i | C |
| T | ur | K |
| H | amiti | C |
| O | at | H |
| S | ara | I |
| P | ear | L |
| I | lia | D |
| T | erro | R |
| A | lsace Lorrain | E |
| L | io | N |

The *Hamitic* tongues comprise the ancient Hieroglyphic language of Egypt, the Coptic, the Ethiopian or Abyssinian, the Libyan, the Hottentot dialect, etc. We cannot accept Hieroglyphic (or Hieratic), a term whose use is limited to the primitive language of *Egypt*, as handed down to us in the monumental inscriptions of that country. Himyaric and himyaritic are also barred, as they are appellative of a language spoken in Arabia, not in Africa.

Correct answers have been received from Robin, Boss, Mater, Dawnlover, Broadwater, Ellice.

No. 10. DISTINGUISHED DOCTORS.

This question has afforded scope for the expression of a great diversity of opinion, even to the extent of the inclusion of the name of Æsculapius, but, unfortunately, the competitor who sent in the name of this fabulous worthy omitted to say whether he regarded him as an English, a Scotch, or an Irish M.D.! The tabulation of the lists sent in has resulted in the following pronouncement of our competitors as to our most distinguished doctors:—

| | |
|---|---|
| Edward Jenner .. .. .. .. .. .. | 14 |
| William Harvey .. .. .. .. .. .. | 13 |
| Sir James Y. Simpson .. .. .. .. .. | 13 |
| John Hunter .. .. .. .. .. .. | 12 |
| Sir Astley P. Cooper .. .. .. .. .. | 12 |
| John Abernethy .. .. .. .. .. .. | 11 |
| William Hunter .. .. .. .. .. .. | 8 |
| Sir Erasmus Wilson .. .. .. .. .. | 7 |
| Thomas Sydenham .. .. .. .. .. | 7 |
| Sir William Fergusson .. .. .. .. .. | 5 |
| Sir Henry Holland .. .. .. .. .. | 5 |
| Thomas Linacre .. .. .. .. .. .. | 5 |
| * * * * * * * | |
| Robert Liston .. .. .. .. .. .. | 5 |

The numbers after the names show the number of "votes" each received. We have been obliged to include thirteen names in the list, owing to the four last being equal.

Best answers have been received from Gip (10), K. M. (8), Letheador (8) Canice (8), Hexameter (8), Ostrich (7), Robin (7), Aralc (7), Mater (7), Ellice (7), Gretta (6), Dawnlover (6), Dolphin (6), May (5), A. M. E. (5), Mrs. Bedingfeld (4), Broadwater (4). The attached numbers show with how many of the tabulated list each correspondent's list agrees.

---

Answers must be addressed to THE PRIZE EDITOR, AMUSEMENTS WITH PROFIT, THE HOSPITAL, Norfolk House, Norfolk Street, Strand, W.C., not later than the *first* post on Friday next. The full name and address must in all cases be given, but a *nom de plume* may be added for publication. Only one side of the paper should be written on. The "Amusements with Profit" Coupon (see advertisement pages) must be enclosed with replies.

